# The role of the applied epidemiologist in armed conflict

**DOI:** 10.1186/1742-7622-1-4

**Published:** 2004-10-07

**Authors:** Sharon M McDonnell, Paul Bolton, Nadine Sunderland, Ben Bellows, Mark White, Eric Noji

**Affiliations:** 1 Centers for Disease Control and Prevention, Atlanta GA, USA; 2 Department of International Health, Boston University School of Public Health, Boston MA, USA; 3 Department of Homeland Security, Washington DC, USA

## Abstract

**Background:**

Applied epidemiologists are increasingly working in areas of insecurity and active conflict to define the health risks, suggest feasible means to reduce these risks and, monitor the capacity and reconstruction of the public health system. In 2001, The Carter Center and the United States Institute for Peace sponsored a conference within which "Violence and Health" was discussed and a working group on applied epidemiology formed. The group was tasked to describe the skills that are essential to effective functioning in these settings and thereby provide guidance to the applied epidemiology training programs.

**Methods:**

We conducted a literature review and consultation of a convenience sample of practitioners of applied epidemiology with experience in conflict areas.

**Results and conclusions:**

The health programs designed to prevent and mitigate conflict are in their early stages of implementation and the evaluation measures for success are still being defined. The practice of epidemiology in conflict must occur within a larger humanitarian and political context to be effective. The skills required extend beyond the normal epidemiological training that focuses on the valid collection and interpretation of data and fall into two general categories: (1) Conducting a thorough assessment of the conflict setting in order to design more effective public health action in conflict settings, and (2) Communicating effectively to guide health program implementation, to advocate for needed policy changes and to facilitate interagency coordination. These are described and illustrated using examples from different countries.

## Introduction

In 2004 it is estimated that there are 95 violent conflicts worldwide [1,2]. The profound consequences to the well-being of communities from these conflicts are disproportionately distributed. Ninety percent of those who die in war are civilians, half are female, and more children will die or be disabled than soldiers [2-9]. Prior to the 1990s, humanitarian assistance in the context of active violence was the domain of emergency medical services; public health and epidemiology focused on refugees and displaced populations [3,6,10,11]. As war became endemic in certain areas, primarily civil and geographically less demarcated, the international public health community was pressured to provide prevention and primary health services to the indigenous population as well [12,13]. The pervasiveness of war and the magnitude of its effects have led public health experts to advocate for directed strategies to prevent and mitigate its effects on communities [2,4,5,12-14]. These strategies are described in the WHO program called Health as a Bridge for Peace (HBP) and have included developing reference and training materials to support health workers in war settings [15-17].

In February 2001 the Carter Center and the United States Institute for Peace (USIP), in collaboration with CARE, Emory University and the Centers for Disease Control and Prevention (CDC), sponsored a meeting on "Violence and Health". The goals of the meeting were to determine the impact of violent conflict on public health and to advise public health training programs on means to enhance the work of public health professionals in settings of violent conflict.

During the meeting a specialty workgroup for "training public health care professionals" was formed and asked to focus on applied epidemiologists by describing their role in conflict prevention, mitigation, and documentation. Applied (or field) epidemiology was initially created by the US Centers for Disease Control and Prevention in the 1950s as a post-doctoral training program [18]. Over time this program has been adopted by more than 35 governments, the World Health Organization (WHO), and numerous schools of public health and non-governmental organizations (NGOs) [19]. The applied epidemiologist is trained to conduct high quality scientific studies and to translate findings into practical, effective public health programs.

Increasingly, applied epidemiologists are recruited to areas of insecurity and conflict to define the health risks, suggest feasible means to reduce these risks, and monitor the capacity and reconstruction of the public health system [20-22]. This trend reflects a growing demand from donors, governments, military, and humanitarian groups for credible information to support the planning and evaluation of health inputs in war. These realities have led to an emerging professional interest within public health on the causes and effects of conflicts [2,13,14,23-25].

## Methods

Because of the increasing demand for applied epidemiologists in conflict, the workgroup leaders reviewed the relevant literature and sought further input from applied epidemiologists and public health practitioners experienced with conflict settings to better understand training needs in this setting. The literature review and discussions focused on the role of applied epidemiologists in violent conflict and what skills are needed to function effectively. The literature review included MEDLINE and Google keyword searches for "war", "conflict", "complex emergencies" and "epidemiology" combined with either "conflict", "disaster" and "refugees". The examination included specific training and reference materials developed to support Health as a Bridge for Peace [16,17]. The leaders expanded their workgroup to include other experts representing the Training in Epidemiology and Public Heal th Network (TEPHINET- a consortium of applied epidemiology training programs in 40 countries), the US Centers for Disease Control and Prevention (CDC), the World Health Organization (WHO), foundations, schools of public health, and numerous private organizations [20,21]. This paper presents the results of the literature review, as well as the answers provided by members of the group at the meeting and subsequently by members of the expanded workgroup via phone interviews about the implications of these recommendations for training field epidemiologists. Examples from the personal experience of the authors are presented and are referenced by giving the individual's initials.

## Results

Applied epidemiologists have an important role in conflict prevention, mitigation, and reconstruction, but to be effective in these circumstances their training must emphasize knowledge about international law, human rights, and complex emergencies [16,17]. In addition, the skills needed must emphasize not only valid collection and interpretation of data but also highlight skills that fall into two general categories: (1) Conducting a thorough assessment of the conflict setting and players, which requires the collection, analysis and interpretation of qualitative and quantitative data to design effective public health action; and (2) Communicating effectively to guide health program implementation, to advocate for needed policy changes and to facilitate interagency coordination. We discuss each of these categories and illustrate each using examples from the experiences of workgroup members in actual conflict settings.

### Conducting a thorough assessment of the conflict setting in order to design more effective public health action in conflict settings

The first contribution of the applied epidemiologist in conflict is to insist that a thorough assessment of the situation be done as soon as possible. Without such insistence the rush to begin programs without adapting interventions to local circumstances will prevail with predictably poor results [2,4,10]. The literature is replete with examples of quick humanitarian action with dire consequences due to poor assessment [10,24,25]. The need to precede the bulk of interventions with valid assessment data might best be summarized in the phrase "Don't just do something, stand there" (and first assess the situation) [20,26,27].

The use of validated qualitative research methods is essential to the processes of building trust and performing high quality assessments. Many workgroup experts suggested including social and behavioural scientists with practical field skills from the beginning; if this is not possible then their techniques for collecting data in the field should be adopted. Using ethnographic skills increases the accuracy and validity of assessments and allows the development of a common vocabulary of key terms for communicating with local people, even when using a translator [28,29]. Despite the inevitable concerns about time, qualitative assessment methods create only a minimal delay in quantitative investigations or services as they require relatively few respondents and can be done in the early stages of response and on a part-time basis. Information gathered in this way makes it less likely that data collected will be misused for political reasons and more likely that samples will be representative and services well targeted [10,26,27].

Once there is agreement that an assessment will be done- either prior to, or (more usually) simultaneously with emergency interventions- the role of the applied epidemiologist is to plan and coordinate the collection of valid and useful data. [19,20,26] The applied epidemiologist brings knowledge of how to achieve this in difficult and resource-poor circumstances by knowing whether and how to make compromises in study design, time, cost, logistics, and security while maintaining acceptable validity. Adequate preparation for the assessment, such as learning the priorities of communities and the language they use to describe health and illness, is invaluable. A good assessment defines the specific context for the health work and assures that the information obtained has a utility that justifies the cost and the risk to health workers and communities. Professionalism and impartiality during the assessment process can set the tone for and facilitate future work. If the role of the epidemiologist in collecting valid and useful data is understood by the community and other relief workers, there is a greater likelihood that the data will be appreciated as being impartial (even if certain groups have an interest in denying this).

The description of previous health programs is one source of useful information for assessments that can be obtained before fieldwork commences. Did previous programs engender trust or suspicion? Did they work, and if so, why? This information can indicate a community's ability to work together or with outside agencies. In addition, if a new program resembles a past program, it will inherit the community's view of the past program, for good or ill. For example, in planning a maternal and child health program in Afghanistan with WHO, we determined that communities that previously had good experiences with girls' schools were much more willing to consider other projects that educated or benefited girls (SMM). Thus, maps from the previous ministries of education and health showing the location of these schools provided initial leads that were followed up by interviews with community members.

Rapid survey methods are most often used to describe the current needs of a population in conflict [3,20]. They aim to describe:

• baseline health status, risks and determinants (e.g. mortality, morbidity, water purity, sanitation, vectors, food, and shelter)

• availability, quality, and use of health services [22]

• access to health care

• the security of a situation [21], and

• changes in the population (e.g. migration) and the conflict (i.e. location of the front lines) [3,21].

Field experience in previous conflicts enables the epidemiologist to know what data are most important to assist in determining risks and designing and assessing interventions in a specific situation. For an assessment to be useful, it must have clear objectives and resist the constant pressure to add more questions [21]. Negotiating questionnaire content among partners can be greatly assisted by agreeing on the final report format, its length and especially, the content of tables and graphs.

Despite the potential shortcomings of rapid surveys, systematically collected data that are appropriately interpreted are superior to anecdote. Additionally, by emphasizing and clearly documenting what sources of data are used to build a conclusion it can be re-evaluated over time. In 1990, patient logs compiled from multiple NGO-supported clinics within Afghanistan revealed that the majority of patient visits were for routine primary care services and not war-related injuries as was assumed. The result was a significant revision of the training curriculum for Afghan medics and physicians in more than 15 non-governmental health service programs (SMM).

Many of the challenges in generating useful, high-quality data can be addressed with innovative data collection methods and judicious interpretation [21]. In conflict settings, a rapid survey of a defined area should take about 1–2 days, and a report of its findings should be provided immediately to decision makers and service providers. For example, an immunization survey in Uganda determined that people in the safest and most easily served areas were un-immunized; it was concluded therefore, that people in hard-to-reach areas would also need immunization. This is an example of the application of the "best case" survey strategy [30].

There are many examples of innovative ways to select a sampling frame in the conflict setting [21]. Global Positioning Systems are increasingly used to identify areas for sampling. Alternatively, satellite imagery can be used to estimate population density and select a geographic area for sampling. The most common approaches for selecting participants in rapid population surveys during conflict are simple random sampling and cluster sampling. The former is easier to analyze and communicate but takes more time in the field. Cluster surveys are more complex to analyze and communicate but involve less field time. More important than which method for sampling is used is to recognize the methods potential biases and how these might limit its applicability [16,29].

In war, obtaining a denominator is very challenging [22,23,26,31,32]. Available population data vary widely in quality, and the movement of persons during conflict can result in inaccurate estimates. The population may be ideologically divided by the conflict, as well as widely dispersed, highly mobile, or in refugee camps [5]. The socio-political circumstances of a population can result in over- or underestimation of its size [11,24,29,31]. For example, women and children may be hidden for protection, or their mobility and access to health and social services severely restricted, resulting in a great underestimation of their numbers. During the civil conflict in Ethiopia in the 1990s, families sequestered young boys to prevent their recruitment as soldiers (SMM). Alternatively, population overestimates may occur when food, drugs, or other resources are rationed [21,29]. Multiple registrations of the same person and not reporting losses through death or departure are ways that families can increase or at least not reduce their resources for use or sale [31]. On the Thai-Khmer border in the 1980s the Khmer Rouge recruited members of other communities to increase their numbers for a census. Addressing these challenges requires an awareness of the forces on the population and the use of measures to accommodate to these forces. In Thailand the UN Border Relief Organization resorted to arriving without notice, calling the community in for registration, and marking those already registered with an indelible stamp to reduce over-counting (PB). In Afghanistan, discussions with community religious leaders revealed that the local mosque recorded households' numbers so as to equitably share food at Islamic holidays. Thus, religious leaders were able to provide an excellent estimate of the number of households and their size (SMM).

Accurate counts of births, deaths, and disease are also affected by social and political instability [31]. Conflict amplifies the risk of chronic and acute health problems while reducing the chance that the affected population will have access to health care [2,13,28]. These increases may go unnoticed, however, because of underreporting from disrupted health systems; demand might even appear to decline [26]. On the other hand, if local authorities believe that reporting disease will increase the flow of resources, such as drugs and equipment, they may inflate these numbers [26]. Rural physicians in the Philippines in 2001 described exaggerating reports of malaria cases in the dry season with the aim to stockpile anti-malarial medications to cover the needs during the malaria season (SMM). Similarly, individuals might feign illness to stock up on medicine if they believe that existing services will be unreliable. For these reasons, data from health services is frequently inaccurate during conflict and should be evaluated in the context of the pressures on the community to survive [10,11,13,21,23,24,29].

The epidemiologist will have to assess the level of access to health-care services for military and non-military personnel, particularly women, children, the elderly, and those with chronic medical conditions [2,11,26]. Defining age-, sex- and cause-specific mortality rates will help assess specific vulnerable groups and determine whether they need special help in accessing services [26,29]. Populations in conflict might suffer overcrowding, poor water and sanitation, and inadequate rations as well as being targeted for violence [2,5,11,13]. All of these can be assessed epidemiologically and determine what interventions should be provided and to whom they should be targeted.

Assessing the data from disease reporting systems is a core function of epidemiologists. If these systems are intact and can describe trends of priority diseases or conditions over time, maintaining them might be worth the effort [21,22]. Typically, however, conflict will interrupt health surveillance activities, including data collection, analysis, interpretation and dissemination, resulting in disease underestimates [2]. As a result, to answer high-priority questions, epidemiologists may need to employ surveillance strategies with less emphasis on routine reporting and more on surveys, sentinel sites, or sentinel populations [21,23,26].

Applied epidemiologists believe that the value of epidemiologic science must also be measured by whether and how it is used to create effective public health action [19]. Applying a public health approach to violence, war, and the factors that initiate and promote them may help identify effective prevention and response programs. For example, knowing the types of weapons used in a conflict makes it possible to predict the types of injuries that will occur [33,34]. At a larger scale, developing systematic predictors of violent conflict may allow earlier intervention, similar to food security and famine early warning programs that monitor the risk of malnutrition. In these programs the emergence of selected behaviours serves as a warning of population risk [35]. The long-term "health" effects on the public from social disruption, violence, poverty, oppression, and torture, are poorly understood, and even less is known about the effectiveness of our programs to address them [36].

Practicing epidemiology in conflict involves working in rapidly changing circumstances and uncertain security. Formal epidemiologic assessments and public health actions in war can only proceed where the environment is "permissive". The security situation for health workers, civilians and programs, as well as community participation and ownership of health programs, must be continuously monitored. Selected parameters that reflect the level of security, participation, demographics and disease should be discussed between partners and security agencies and re -evaluated frequently [2]. Health agencies must demand a level of security to function; however, their conduct on the ground may influence the extent to which they are afforded protection by local communities.

Acute and chronic war settings, while superficially similar, represent different challenges to the epidemiologist and can significantly affect the type of intervention selected [23]. In wars of short duration – such as Kuwait after the first Gulf war – the coping mechanisms of the population (for example, how they maintain their health and where they go for health services) are still based on peacetime conditions [32,37]. The epidemiologist may be able to focus on the pre-existing public health and surveillance system, which is likely to be partially intact for at least some parts of the population [26]. The epidemiologist can work with those local health workers and officials who are still in place in order to understand the local situation, define and prioritize health problems, and reestablish basic services in a form acceptable to local people.

A chronic war setting (e.g. Afghanistan) is more challenging [13,23]. Knowledge of the pre-war situation is still useful, but often little is left of the previous public health system, and people will have developed new coping mechanisms to deal with prolonged war. The epidemiologist must spend more time investigating the current situation and assist appropriate agencies to plan and build new systems that meet current needs. Because of the lack of predictability, security and centralized authority, all plans and programs will need to be tested at a smaller scale before expansion to a larger population. Building systems will take longer due to the lack of pre-existing resources and trained personnel. In Iraq, although the current conflict is "acute", it also follows ten years of sanctions after a previous war and a major change in leadership. Although much of the pre-2003 health infrastructure remains, the goal is not to replicate the old system with its poor supplies and limited access.

### Communicating effectively to guide health program implementation, advocate for needed policy changes and facilitate interagency coordination

All aspects of communication are more difficult during conflict [10,26]. Pre-existing infrastructure may have been destroyed and, if not, it will be part of the struggle for power and control. Practical solutions to communication needs require flexibility and a higher proportion of the budget than is needed in more stable and secure situations. Radio, satellite, and cellular phones have advantages that make them very useful, but because of their cost these technologies are often out of reach for many programs. Aid organizations should collaborate to solve communication problems and share resources; donors need to encourage and reward this by allowing local coordination of grant funds to reduce redundancy. Included in communication skills are the abilities to facilitate inter-agency coordination and collaboration and to work with multiple constituencies. Coordination among program and donors offers an opportunity to eliminate waste and to encourage equity. In Uganda in 2001, local police allowed public health workers in isolated locations to use their radios to communicate surveillance information on outbreaks to the Ministry of Health (Personal communication P. Nsubuga 2001).

Ongoing consultation with communities is necessary for the integrity of programs and to monitor security. Epidemiologists can improve the quality of the information they receive and disseminate by developing alliances with partners who have a communication infrastructure in place- and people travelling into areas that are restricted or insecure. For example, contacting agencies that work in agriculture, health care, education, land-mine removal, and food provision about sharing or coordinating field staff that monitor and supervise projects can be helpful. Jointly planning the efficient use of field staff from all types of programs using checklists, and other tools to help the non-technical visitor be able to bring back useful information and support local communities can help these communities feel less isolated, reduce waste and increase the data sources about health programs and problems.

Workgroup members suggested that journalists may be potential sources of useful information, insofar as they have access to restricted areas. They may be able to:

• report on health-related activities or risks (e.g., immunizations, outbreaks, and unexpected behaviour in selected populations),

• provide reliable communication with all sides and help establish ceasefires for the provision of health services [38], and

• help control rumours by defining means to check information early and responding to misinformation quickly [22,26].

The different goals of the media and public health and the rapid turnover of journalists, however, may limit useful cooperation.

Epidemiologists in Colombia's Centro de Referencia Nacional sobre Violencia (CRNV) used national media coverage to highlight political homicides. They published information regarding deaths along one particular river in Colombia to raise awareness of the level of violence occurring there. This data-based approach drew less political resistance while focusing public attention on the deaths [39,40].

Effective communication strategies should target policy-makers from local to international levels. Epidemiologists can be an effective voice for public health if they maintain their credibility with policy-makers. Sometimes the most important role for the epidemiologist is to act as a witness to describe the local situation to an international audience of policy-makers and to advocate for action [2,10,27,41,42]. Using a public health approach the epidemiologist can describe the realities of war, its effects on individuals and communities, and the consequences of certain types of weapons, warfare and humanitarian approaches. We must think of war not as a natural consequence of life but as a preventable tragedy with multiple long-lasting implications. The decision to use violence must be examined openly and not sanitized [43].

A less rapid but possibly more respected method for bringing awareness of local health and social issues to national and international levels is publishing research in credible journals. Peer-reviewed work can be a tool to facilitate negotiations between governments, NGOs and communities, and to influence international and national level policy-makers.

Effective advocacy for public health includes the ability to use communication skills such as advocacy, consensus development, and negotiation to promote population health to policy-makers [23,26,28,42,44]. As much as possible, messages should be communicated directly to the local population rather than through political leaders or another entity, as the messages may be tainted or obscured by differing agendas. For example, in the Thai border refugee camps epidemiological data were interpreted and then translated into health messages that were communicated by program staff via megaphones while travelling around the community (PB). In 2003 the US military personnel and reservists in Iraq were responsible for providing humanitarian aid and direct interactions with civil authorities; a role for which they had no training (EJN). In addition, because of the military association, the information gathered was considered inherently biased and dismissed.

During conflict, health and information services that were once unified under a single health ministry can become fragmented with parts falling under the aegis of government, those resisting the government, or nongovernmental organizations [23,31]. Access to health care and humanitarian resources can be important in political battles at the local level [26]. Each party in a conflict wants control of hospitals, health workers, food, and medications so as to be able to offer them to the community, thus enhancing their credibility, and to aid its military efforts (SMM). In Colombia, some communities have lived in conflict for > 40 years despite local, national, and international efforts to find solutions to the chronic violence. Epidemiologists there described attempts to ameliorate animosities by involving local constituencies in health activities: the Church, NGOs, elected officials, and other leading organizations or personalities in the community (Personal communication Jorge Jara 2002). Local rather than national health authorities chose priorities, from outbreak investigations and interventions to data analysis to surveillance of violence and influenza. Through this process trust was built between the Colombia National applied epidemiology program and the local health authorities, thereby assuring access to the population that transcended partisanship. Parties on all sides of a political/military issue collaborated on a community health project, an unprecedented step.

Many workgroup members felt that negotiation skills were essential when working with multiple aid groups or the main parties in the armed conflict. For example, consensus building is useful when the health problems are not well defined and diverse groups must collectively identify and solve them. At the simplest level this includes the skill of running meetings and enhancing a group's utility and the understanding that disagreement might not necessarily represent failure. The goal is to help all parties understand that improving health meets the interests of all groups and to develop complimentary strategies. Too often decisions are rushed and participants pushed aside, leading to unsuccessful outcomes and future difficulties working together [44]. When disagreements are so deep that one or both sides will not accept any solution that benefits the other side, negotiation will be fruitless. Currently, in Iraq, addressing the emergency and reconstruction needs of the Iraqi people through a collaborative process that involves the full range of stakeholders – Iraqi, Coalition, UN, NGOs, Civil Affairs, Donors – remains out of reach (EJN).

To facilitate the coordination of health services, the epidemiologist needs to be familiar with the many agencies working in the target area, including governmental, nongovernmental, and United Nations agencies. The diversity of groups may make it difficult to establish a shared view of the situation, much less a collaborative plan of action. Additional obstacles to coordination may stem from competition for resources, lack of information or true differences in their philosophy about humanitarian aid and their ability to deliver it. Donors in particular can create a powerful force for coordination if they agree on priorities and processes. International donors can influence policy-makers by providing resources and technical support conditional on their commitment to the resolution of violent conflict [4]. It is the job of the applied epidemiologist to supply compelling credible information for these decisions. By providing credible information and establishing it as the foundation for action the epidemiologist can greatly assist coordination. Numerous workgroup members mentioned that being able to provide published medical and public health literature to government officials, donors and NGOs facilitated their efforts to coordinate agencies and to build consensus by providing an impartial standard based on scientific data [32]. One implication is that the epidemiologist needs rapid access to international literature, possibly via the Internet, even in war zones.

## Summary

Applied epidemiologists can use their skills and position to promote positive population health policies and programs to address inequities that exacerbate conflict and violence [1,2,4]. In addition, credible on-site information can reduce the waste and harm of poorly planned humanitarian assistance [9,10,23-25]. Epidemiologists' work among policy-makers and the public uniquely positions them to communicate the effects of war, advocate for the population and assist in the reconstruction of health systems [9,15,23]. As impartial agents, epidemiologists can promote dialogue between conflicting parties, influence public opinion, facilitate projects that require cooperation, and coordinate multilateral responses to health and humanitarian needs [13,22,23]. Banning landmines, creating days of tranquillity for immunization, and surveillance of homicide resulting in limiting firearms in Colombia are examples of these ideas at work [38-40,44,45].

Providing health resources, including epidemiological support, should be part of a much larger diplomatic and humanitarian strategy. In fact, when a larger framework and commitment are missing, the health activities are unlikely to result in lasting changes and only increase the risks to the entire population. [26,47]. Epidemiologists are not diplomats nor should they be gratuitously sent into conflict situations as humanitarian band-aids. In addition, there are real risks that scientific information, meant to improve health, might be usurped for political aims rather than humanitarian purposes [14,27]. For example, in Colombia the data from health surveys undertaken with the best of intentions were used to locate populations for military action (Personal communication G. Suarez, 2002).

Epidemiologists need to advocate for focused field research on conflict resolution and violence prevention, and to evaluate the success of health programs in conflict [2,25,36]. It is no longer practical to conceptualize complex humanitarian emergencies like war into distinct phases [2]. In reality the settings for disasters, refugees, active conflict, and anxious peace are in constant transition. Defining and evaluating social and individual risk markers for violence may facilitate setting up early warning systems [46]. The reluctance to evaluate health programs in war stems from the assumption that doing anything is better than doing nothing and perhaps that good evaluation is too difficult. These flawed assumptions preclude progress and condemns us to rigid approaches resulting in the continued use of interventions of unknown effectiveness [13]. To paraphrase a military saying, "we are always treating the health problems of the last war".

Well-documented epidemiologic methods to assess conflict settings combined with ongoing re-assessment of the assumptions for interventions will greatly facilitate health program evaluation. In addition, measures of success for health programs need to reach much further into cultural competence, economic development, and social well-being. Based on the results of these studies more effective and cost-effective methods for public health response could be implemented in the future. With the number and intensity of current armed conflicts there is even greater urgency to begin this work.

The knowledge and skills described here are not typically part of epidemiology training. Yet they do fit with the epidemiologist's mission of gathering data and using it to improve the lot of populations. The materials developed to date to train health personnel working in war zones need more specificity and case examples. However, whether epidemiologists are able to respond to war largely depends on the availability of appropriate additional training and modelling by teachers and practicing epidemiologists [2,23]. Education programs designed for these goals would need to expand knowledge of human rights and international law, qualitative research methods, innovative ways to gather reliable population information during conflict, and effective methods to communicate this information. The working group strongly emphasized that these skills cannot be imparted solely in didactic courses, and training must include simulations with supervised field practice.

However well-intentioned the science and programs of epidemiology, we must be vigilant that epidemiology benefits public health, that it is not used to contribute to the prolongation of conflict and that it does not become part of it [13,22-25,33]. Insofar as epidemiologists influence decision-makers, we must strive to do so by adapting our work to the goals of peace through every means at our disposal. [33,42].

We should all strive for a time when, through the efforts of public health workers and others, war too will be eliminated.

*- Jimmy Carter *[2]
